# Supplementary material for: Region-specific defect engineering of Bi2W1-xO6-γ induces nanoscale electric fields and surface active-sites for enhanced visible-light oxidation of salt-lake flotation agents
Source: Nat Commun. 2025 Nov 28;16:11296. doi: 10.1038/s41467-025-66466-5 (PMC12722310; doi:10.1038/s41467-025-66466-5)
Supplement: Supplementary file 1 — Supplementary Information [file 41467_2025_66466_MOESM1_ESM.pdf]

Supplementary information

Region-Specific Defect Engineering of $Bi_2W_{1-x}O_{6-\gamma}$ Induces Nanoscale Electric Fields and Surface Active-Sites for Enhanced Visible-Light Oxidation of Salt-Lake Flotation Agents

Liang Ma [1, 2], Siyuan Zhang [1, *], Haining Liu [1], Chunyan Wang [1, 2], Zhongmei Song [1, 2], Wenjie Han[1], Mingzhe Dong[1], Jungang Hou [3, *], Weidong Shi [4, *], Xiushen Ye [1, *]

[1] *Key Laboratory of Green and High-end Utilization of Salt Lake Resources, Qinghai Provincial Key Laboratory of Resources and Chemistry of Salt Lakes, Qinghai Institute of Salt Lakes, Chinese Academy of Sciences, Xining 810008, China*

[2] *University of Chinese Academy of Sciences, Beijing 101408, China*

[3] *State Key Laboratory of Fine Chemicals, Frontiers Science Center for Smart Materials Oriented Chemical Engineering, School of Chemical Engineering, Dalian University of Technology, Dalian 116024, China.*

[4] *College of Environmental and Chemical Engineering, Jiangsu University of Science and Technology, Zhenjiang 212003, China.*

E-mail addresses: siyuanzh@isl.ac.cn (Siyuan Zhang), jhou@dlut.edu.cn (Jungang Hou), swd1978@ujs.edu.cn (Weidong Shi), yexs@isl.ac.cn (Xiushen Ye).

Supplementary Table of content

Text S1 Samples synthesis process..1
Results and Discussion 4
1. SEM image..4
2. Nitrogen absorption-desorption isotherms 5
3. TEM image 6
4. High resolution (HR)-TEM image..7
5. EDS elemental mapping profile..8
6. Average values of HRTEM EDS elemental line scanning of BWO-ES 9
7. EDS spectra..10
8. AFM image 11
9. XPS spectra of Bi element in BWOs..12
10. Sputtering time-dependent XPS spectra of the W 4f in BWO 13
11. Sputtering time-dependent XPS spectra of the O 1s in BWO..14
12. Sputtering time-dependent XPS spectra of the Bi 4f in BWO 15
13 Sputtering time-dependent XPS spectra of the Bi 4f in BWO-ES....................................16
14. WT-EXAFS of BWO and EXAFS fitting parameters of BWO and BWO-ES..................17
15. Positron lifetime spectrum test data..18
Text S2 The process of photocatalytic degradation of ODA..19
Text S3 The process of photocatalytic degradation of DMP..20
16. Degradation kinetics fitting curve 21
17. Conversion of N element in ODA degradation process 22
18. TOC degradation curve..23
19. Infrared spectroscopy of catalyst surface in the degradation process of ODA..................24
20. Quenching experiment of octadecylamine degradation 25
21. Quenching experiment Rate fitting..26
22. EPR determination of ·OH generation 27
23. Recycling stability of BWO-S and BWO-E..28
24. Recyclability of photodegradation of ODA 29
25. Comparison of degradation performance of ODA and DMP between BWO-ES and reported materials..30
26. UV-vis diffuse reflectance spectra..31
27. Calculated DOS 32
28. PL spectra..33
29. The surface photovoltage spectra 34
30. Model parameters of the photocatalysts based on EIS results 35
31. VB-XPS spectra of prepared materials..36
32. The surface charge separation efficiency histogram 37
33. KPFM spectra 38
34. The transient photocurrent response spectra 39
35. Calculation of dipole moment 40
36 Toxicity analysis of ODA and DMP degradation products 41
Text S4 Chemicals 43

Text S5 Crystal structure and morphology testing ... 44
Text S6 Elemental composition and valence analysis ... 46
Text S7 Photoelectrochemical property testing ... 47
Text S8 Investigation of degradation process ... 49
Text S9 Calculation of energy band structure ... 51
Text S10 Calculation of adsorption energy ... 52
Text S11 Finite element simulation of surface local electric field intensity in materials ... 53
Text S12 Calculation of dipole moment ... 54
References ... 55

Text S1 Samples synthesis process

(1) Bi_2WO_6

Weigh 1.94 g of $Bi(NO_3)_3{\cdot}5H_2O$, 0.658 g of $Na_2WO_4{\cdot}2H_2O$, and 0.12 g of cetyltrimethylammonium bromide (CTAB), dissolve them in 60 mL of water, sonicate at room temperature for 0.5 h, stir magnetically for 4 h to completely dissolve the reagents, and then transfer them to a 100 mL polytetrafluoroethylene reaction kettle for hydrothermal reaction at 180°C for 18 h. After cooling to room temperature, wash several times with anhydrous ethanol and deionized water respectively, then vacuum dry at 60°C for 10 h. Sample marked as BWO.

(2) $Bi_2W_{1-x}O_6$

Weigh 1.94 g of $Bi(NO_3)_3{\cdot}5H_2O$, 0.658 g of $Na_2WO_4{\cdot}2H_2O$, 0.12 g of cetyltrimethylammonium bromide (CTAB), and NaOH (with masses of 0.0080 g, 0.0125 g, 0.0250 g, 0.080 g, and 0.100 g, respectively) and dissolve them in 60 mL of water. Ultrasonic treatment at room temperature for 0.5 h, magnetic stirring for 4 h to completely dissolve the reagent, and then transfer it to a 100 mL polytetrafluoroethylene reaction kettle for 18 h of hydrothermal reaction at 180°C. After cooling, rinse several times with anhydrous ethanol and deionized water, then vacuum dry at 60°C for 10 h. The XRD pattern and photo of the material are shown in **Supplementary Fig. S1**.

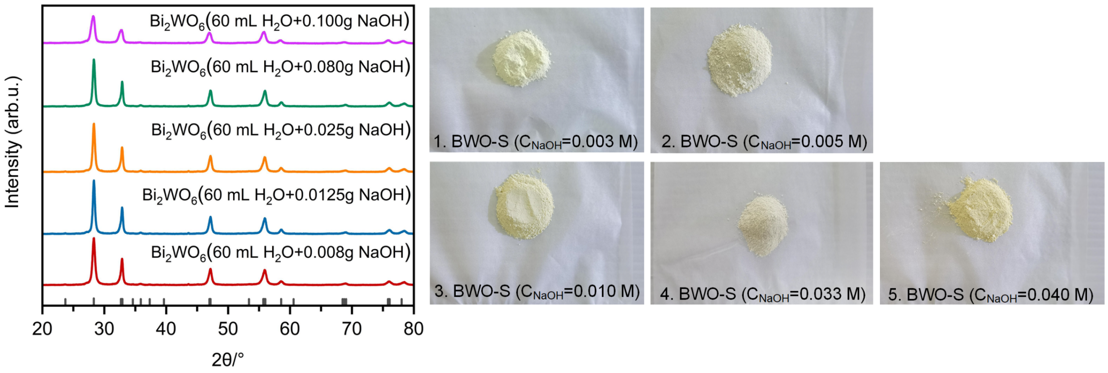

Supplementary Fig. 1 XRD pattern and images of BWO-S. The left and right part are the XRD patterns and images of synthesized BWO-S, respectively.

(3) Bi_2WO_{6-y}

Weigh 1.94 g of $Bi(NO_3)_3{\cdot}5H_2O$, 0.658 g of $Na_2WO_4{\cdot}2H_2O$, and 0.12 g of cetyltrimethylammonium bromide (CTAB) and dissolve them in 10 mL, 30 mL, 50 mL, and 60 mL of ethylene glycol (EG), respectively. Then, add 50 mL, 30 mL, 10 mL, and 0 mL of deionized

water in sequence to make the total volume of the mixed solution 60 mL. Afterwards, sonicate at room temperature for 0.5 h, stir magnetically for 4 h to completely dissolve the reagent, and then transfer it to a 100 mL polytetrafluoroethylene reaction vessel for hydrothermal reaction at 180°C for 18 h. After cooling, wash several times with anhydrous ethanol and deionized water, and vacuum dry at 60°C for 10 h. The XRD pattern and photo of the material are shown in **Supplementary Fig. 2**.

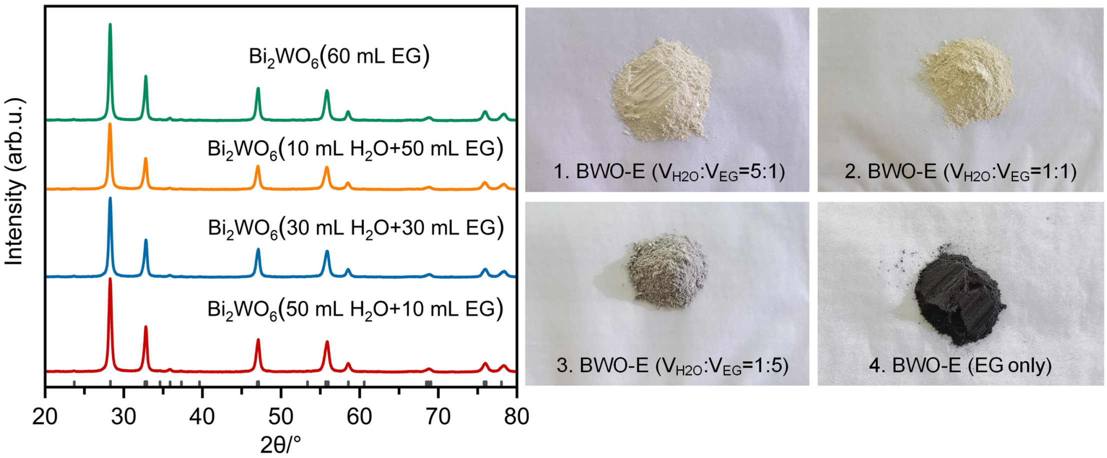

Supplementary Fig. 2 XRD pattern and images of BWO-E. The left and right part are the XRD patterns and images of synthesized BWO-E, respectively.

(4) $Bi_2W_{1-x}O_{6-y}$

Weigh 1.94 g of Bi $(NO_3)_3 \cdot 5H_2O$, 0.658 g of $Na_2WO_4 \cdot 2H_2O$, 0.12 g of cetyltrimethylammonium bromide (CTAB), and 0.08 g of NaOH and dissolve them in 60 mL of ethylene glycol (EG). Ultrasonic treatment at room temperature for 0.5 h and magnetic stirring for 4 h until the reagents are completely dissolved. Then transfer the mixture to a 100 mL polytetrafluoroethylene reaction kettle and react hydrothermal at 180°C for 18 h. After cooling, wash several times with anhydrous ethanol and deionized water, and vacuum dry at 60°C for 10 h.

Subsequently, the crystal structures of different materials prepared were characterized by XRD, and Bi_2WO_6 materials were successfully prepared by different preparation methods. The elemental ratios of the materials were tested by XRF (**Supplementary Table1**), and the structure showed that the concentration of W defects could be adjusted by changing the dosage of NaOH (S), and the concentration of O defects could be adjusted by changing the ratio of ethylene glycol to deionized water.

Supplementary Table1 XRF atomic ratios of $Bi_2W_{1-x}O_6$ and Bi_2WO_{6-y}.

Sample	Method	Bi_2O_3 (wt%)	WO_3 (wt%)	$n_{Bi}:n_W:n_O$
$Bi_2W_{1-x}O_6$ (I)	C_{NaOH}=0.003 M	66.78	33.19	2.00:1.00:6.01
$Bi_2W_{1-x}O_6$ (II)	C_{NaOH}=0.005 M	67.22	32.78	2.00:0.98:5.96
$Bi_2W_{1-x}O_6$ (III)	C_{NaOH}=0.010 M	67.90	33.07	2.00:0.95:5.90
$Bi_2W_{1-x}O_6$ (IV)	C_{NaOH} =0.040 M	69.31	30.64	2.00:0.89:5.72
Bi_2WO_{6-y} (I)	$V_{EG}:V_{H_2O}$=1:5	67.01	32.99	2.00:0.99:5.97
Bi_2WO_{6-y} (II)	$V_{EG}:V_{H_2O}$=1:1	67.38	32.62	2.00:0.97:5.92
Bi_2WO_{6-y} (III)	$V_{EG}:V_{H_2O}$=5:1	67.60	32.40	2.00:0.96:5.84

Results and Discussion

1. SEM image

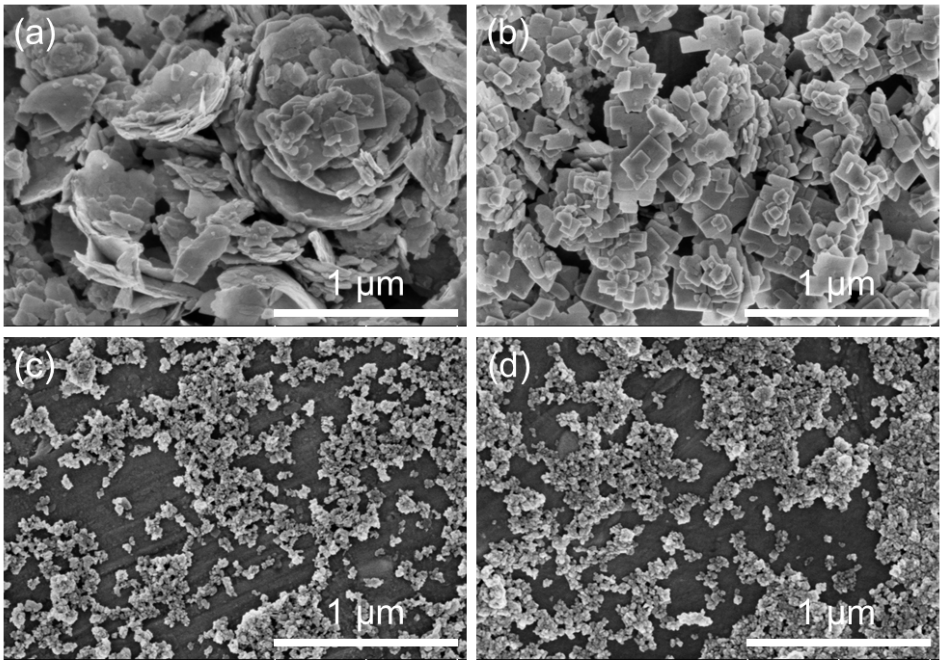

Supplementary Fig. 3 SEM images. SEM images of (a) BWO, (b) BWO-S, (c) BWO-E and (d) BWO-ES.

2. Nitrogen absorption-desorption isotherms

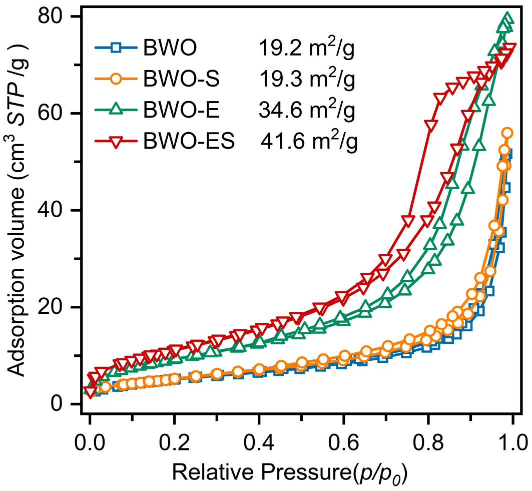

Supplementary Fig. 4 Nitrogen absorption-desorption isotherms. Nitrogen adsorption-desorption isothermal of samples.

3. TEM image

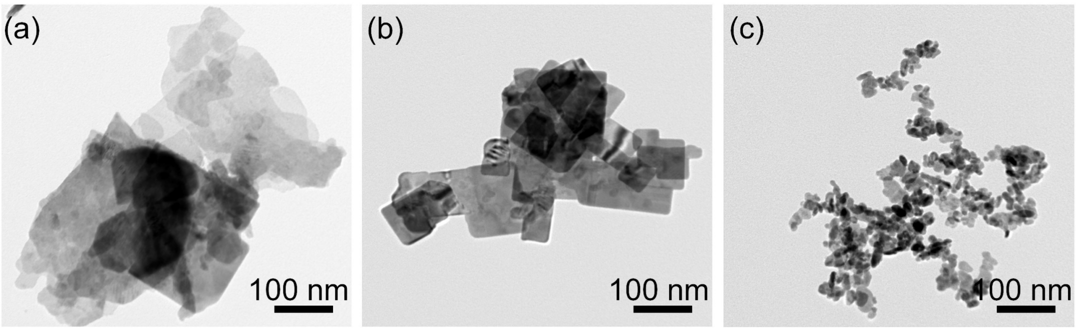

Supplementary Fig. 5 TEM images. TEM images of (a) BWO, (b) BWO-S, and (c) BWO-E.

4. High resolution (HR)-TEM image

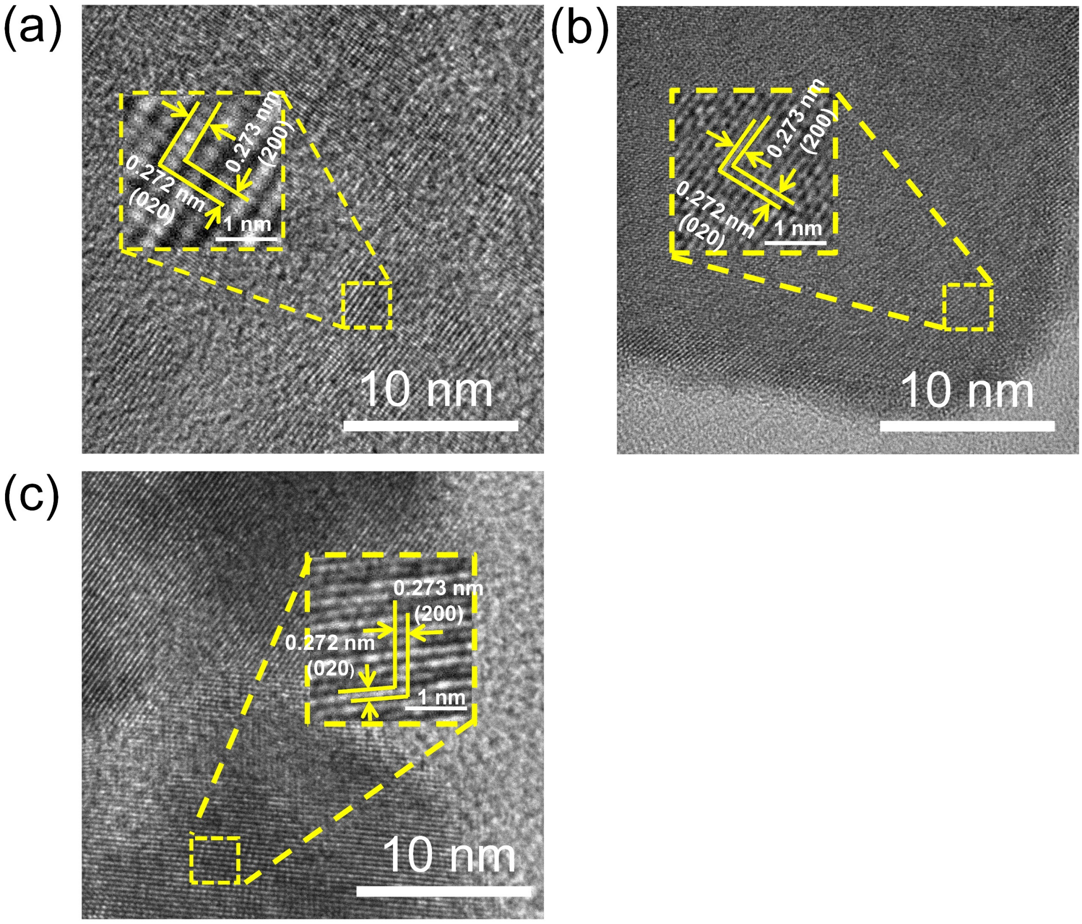

Supplementary Fig. 6 High resolution-TEM images. HR-TEM images of (a) BWO, (b) BWO-S, and (c) BWO-E.

5. EDS elemental mapping profile

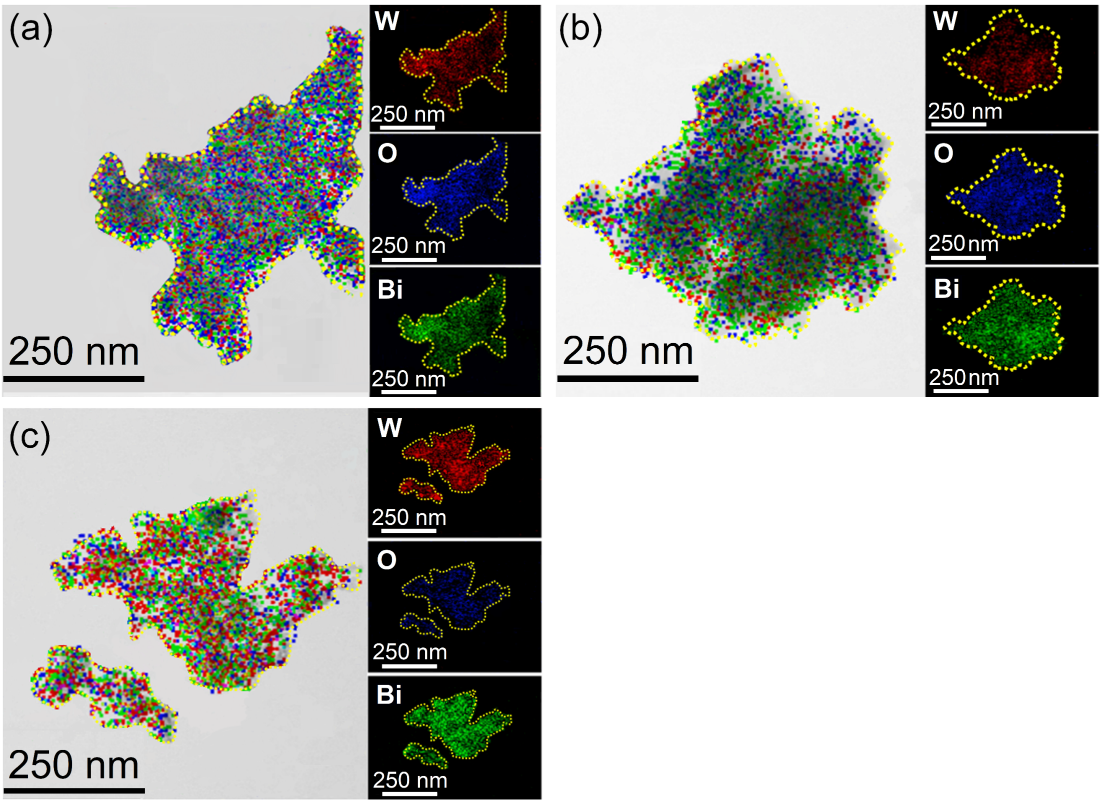

Supplementary Fig. 7 EDS elemental mapping profiles. EDS elemental mapping profiles of (a) BWO, (b) BWO-S, and (c) BWO-E.

6. Average values of HRTEM EDS elemental line scanning of BWO-ES

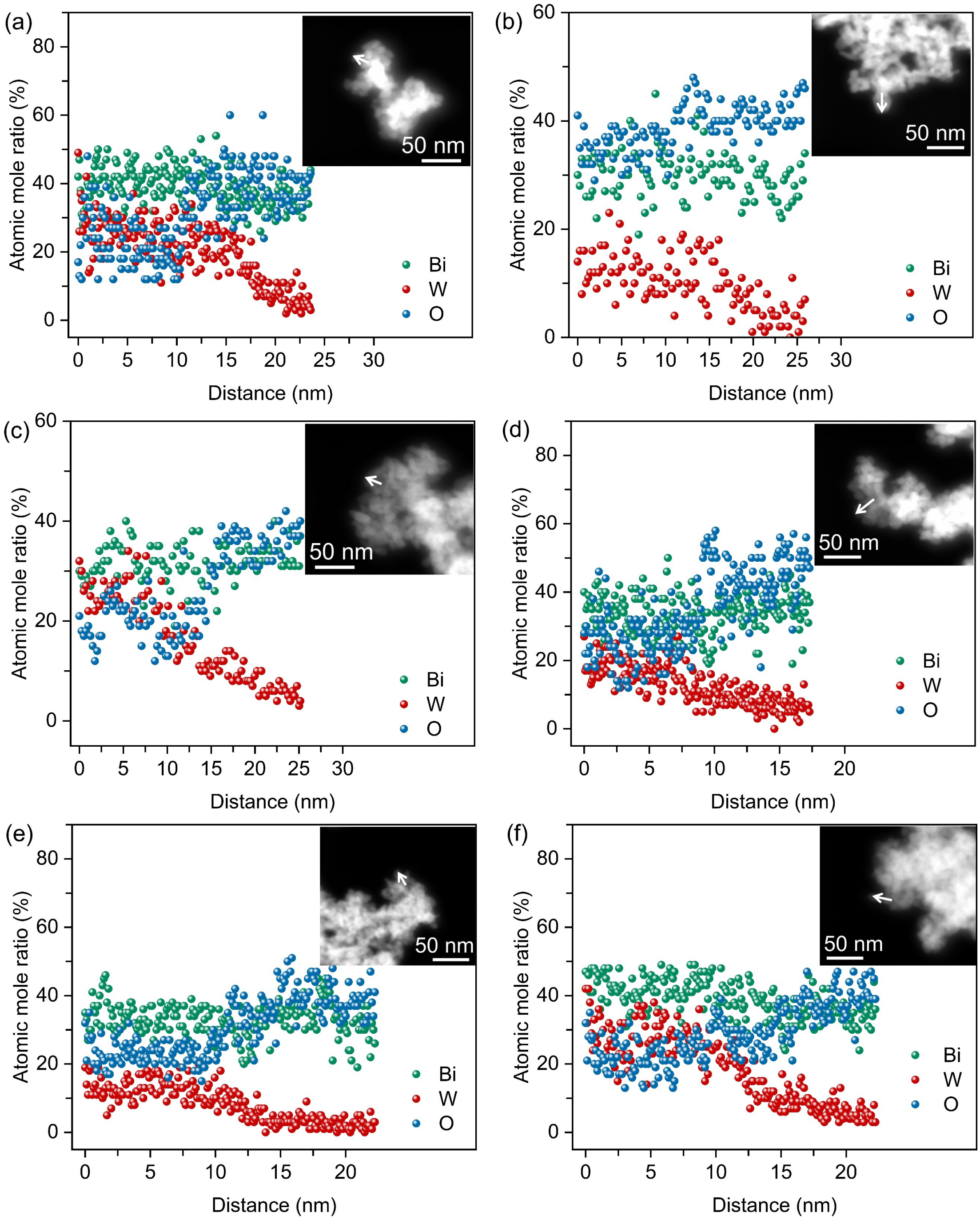

Supplementary Fig. 8 HRTEM EDS elemental line scanning. Average values of HRTEM EDS elemental line scanning along the white arrow in the inset of HRTEM images of BWO-ES.

7. EDS spectra

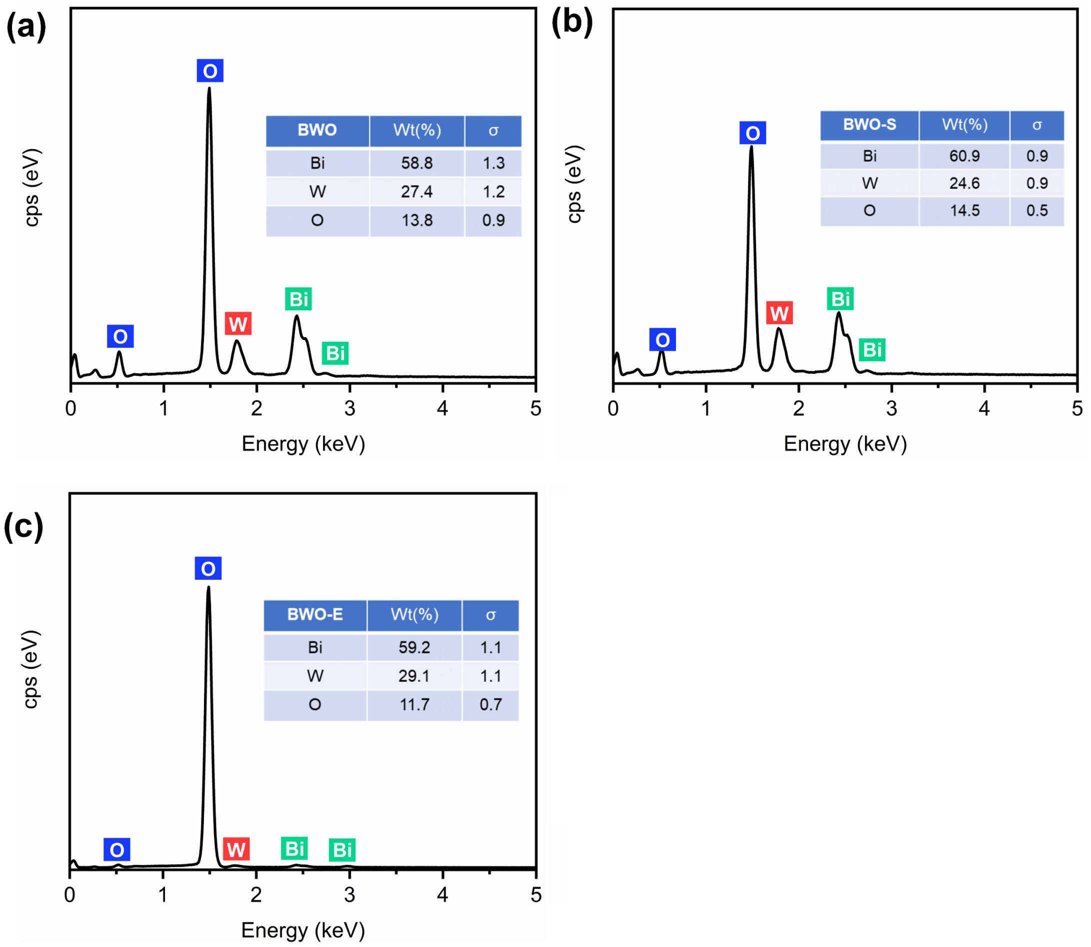

Supplementary Fig. 9 EDS spectra. EDS spectra of (a) BWO, (b) BWO-S, and (c) BWO-E

8. AFM image

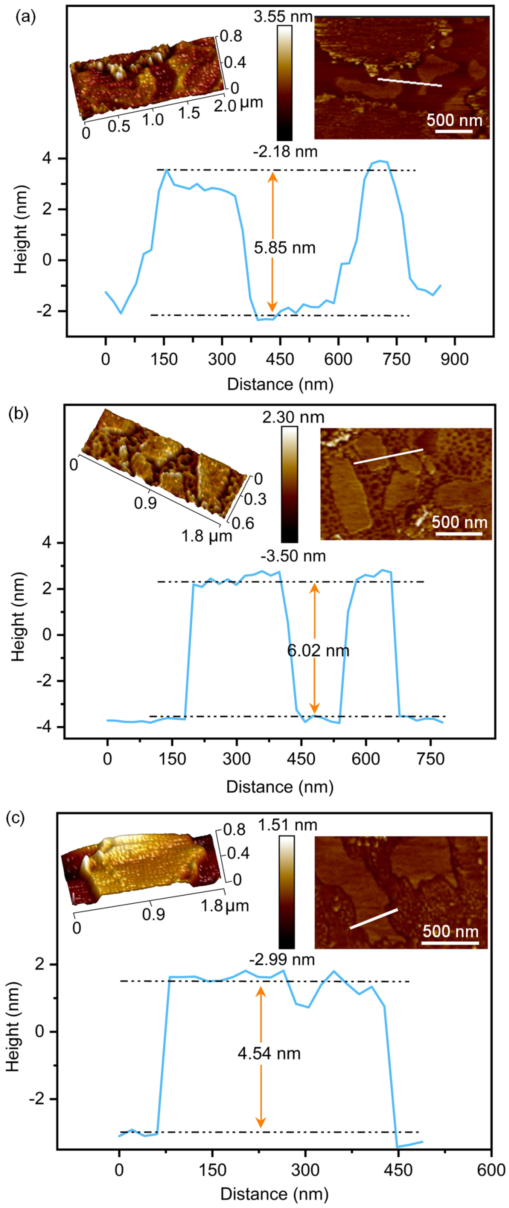

Supplementary Fig. 10 AFM image. AFM image and the corresponding height profile of (a)BWO, (b)BWO-S, and (c)BWO-E.

9. XPS spectra of Bi element in BWOs

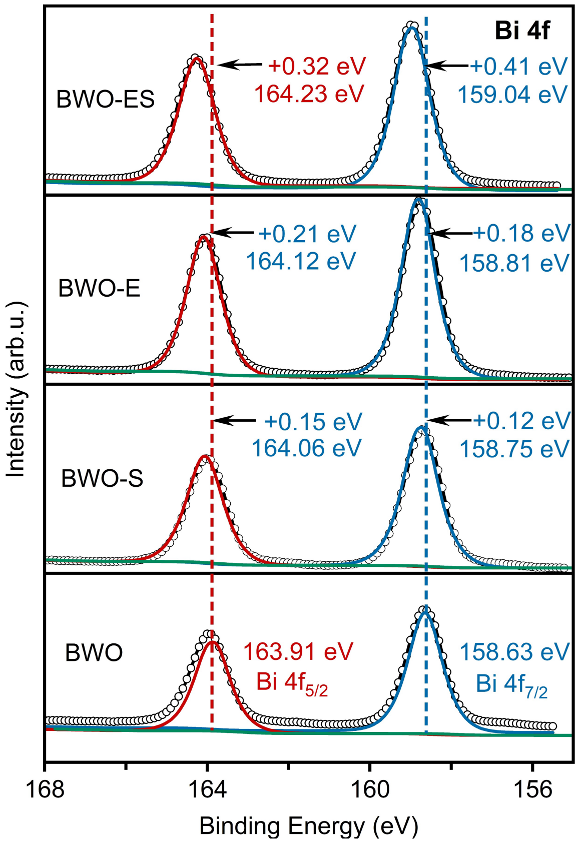

Supplementary Supplementary Fig. 11 XPS spectra. XPS spectra of Bi element in prepared materials.

10. Sputtering time-dependent XPS spectra of the W 4f in BWO

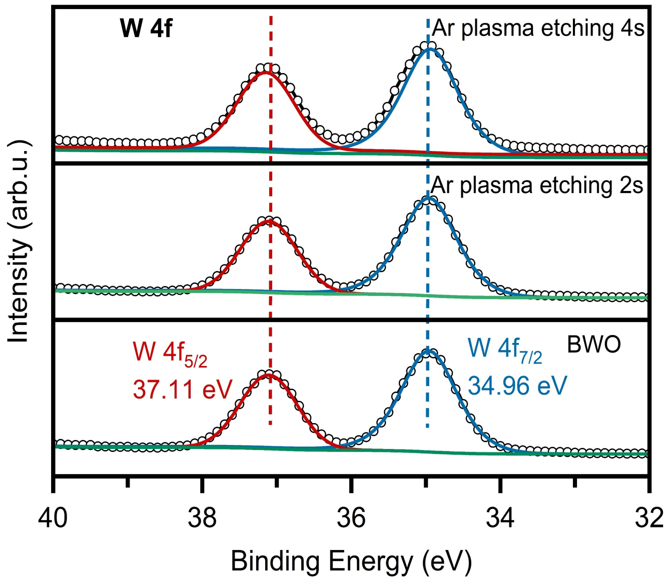

Supplementary Fig. 12 Etching XPS spectra. Sputtering time-dependent XPS spectra of the W 4f state recorded from BWO.

11. Sputtering time-dependent XPS spectra of the O 1s in BWO

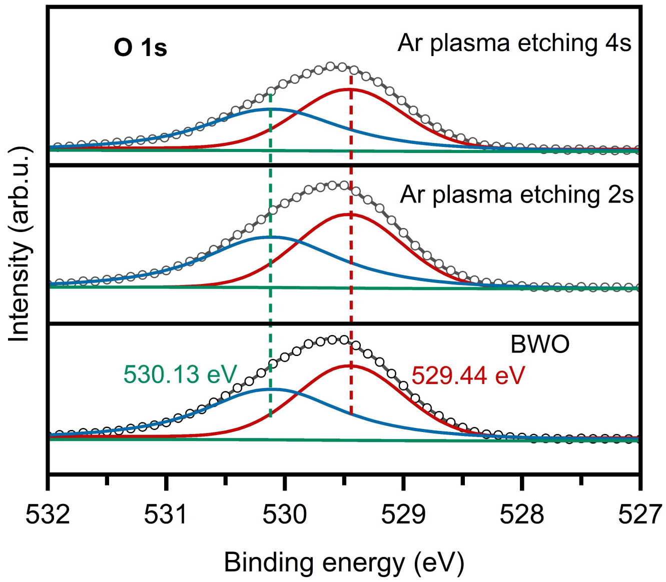

Supplementary Supplementary Fig. 13 Etching XPS spectra. Sputtering time-dependent XPS spectra of the O 1s state recorded from BWO.

12. Sputtering time-dependent XPS spectra of the Bi 4f in BWO

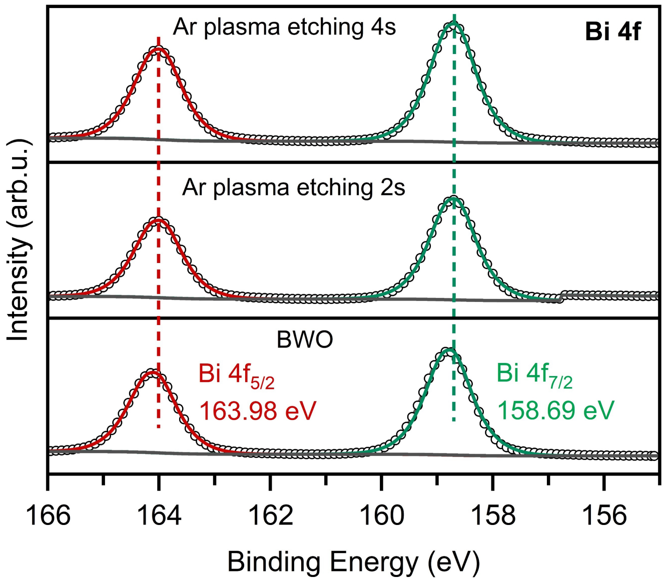

Supplementary Fig. 14 Etching XPS spectra. Sputtering time-dependent XPS spectra of the Bi 4f state recorded from BWO.

13 Sputtering time-dependent XPS spectra of the Bi 4f in BWO-ES

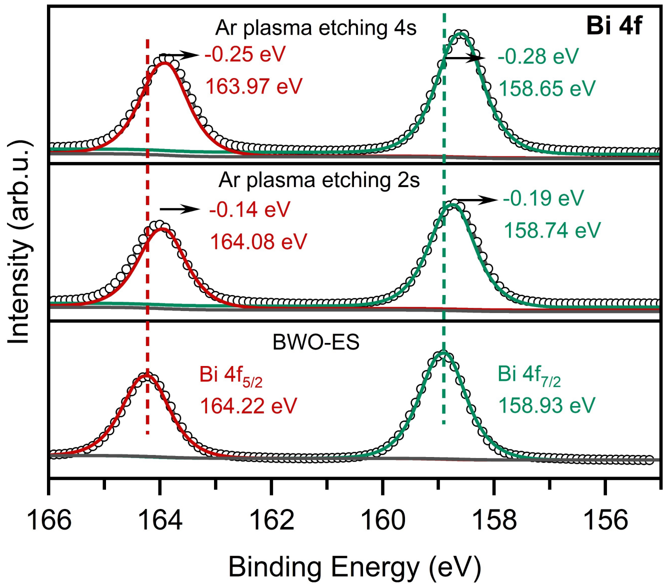

Supplementary Fig. 15 Etching XPS spectra. Sputtering time-dependent XPS spectra of the Bi 4f state recorded from BWO-ES.

14. WT-EXAFS of BWO and EXAFS fitting parameters of BWO and BWO-ES

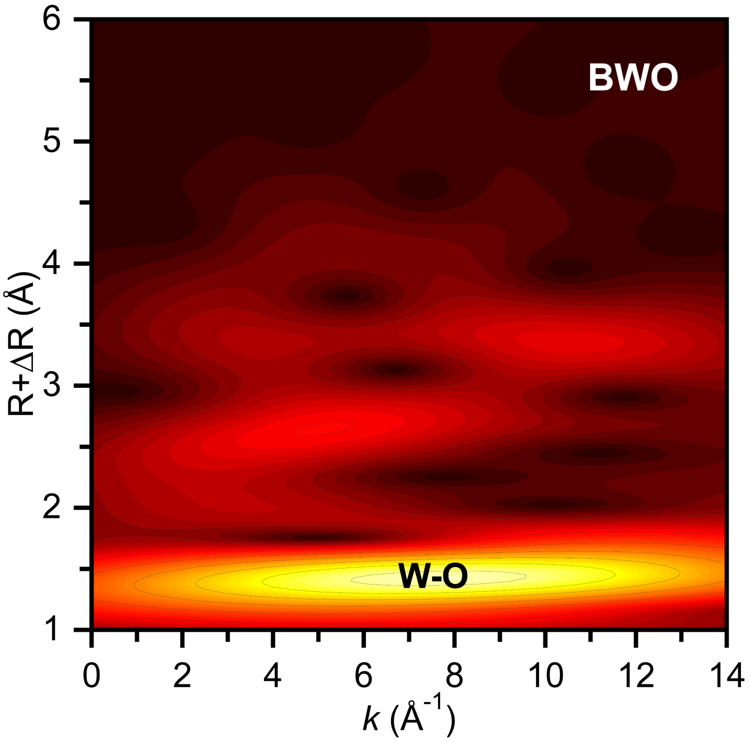

Supplementary Fig. 16 WT-EXAFS of BWO. The wavelet transform extended x-ray absorption fine structure for the BWO sample.

Supplementary Table 2 EXAFS fitting parameters at the W L-edge for W foil, BWO and BWO-ES.

Sample	Path	N	R（Å）	σ^2（×10^{-3} $Å^2$）	ΔE_0（eV）	R-factor
W foil	W-W	8	2.72(0.01)	2.95(0.80)	7.14(1.53)	0.009
		6	3.13(0.01)	2.95(1.12)		
BWO	W-O	3.32(0.58)	1.78(0.02)	3.42(1.85)	2.61(2.68)	0.014
BWO-ES	W-O	3.18(0.48)	1.77(0.01)	2.72(1.51)	2.21(2.32)	0.011

N, coordination number; *R*, distance between absorber and backscatter atoms; σ^2, Debye-Waller factor to account for both thermal and structural disorders; ΔE_0, inner potential correction; *R* factor indicates the goodness of the fit.

S_0^2 was fixed to 0.80, according to the experimental EXAFS fit of W foil by fixing CN as the known crystallographic value. A reasonable range of EXAFS fitting parameters: $0.600 < S_0^2 < 1.000$; $CN > 0$; $\sigma^2 > 0$ $Å^2$; $|\Delta E_0| < 15$ eV; R factor < 0.02. [1]

15. Positron lifetime spectrum test data

Supplementary Table 3 Positron lifetime spectrum test data.

Sample	Intensity(I_1)	τ_1(ps)	Ratio(%)	Intensity(I_2)	τ_2(ps)	Ratio(%)	Intensity(I_3)	τ_3(ns)	Ratio(%)	$\tau_{ave.}$(ps)
BWO	9755.03	172.9	13.88	9145.91	412.6	31.07	3823.59	1.75	55.05	1114.3
BWO-ES	10781.23	188.4	15.92	9605.62	385.3	29.01	3689.12	1.91	55.07	1190.9

The average positron lifetime ($\tau_{ave.}$) was calculated as follows:

$$\tau_{ave.} = \frac{\sum_{n=1}^{3}(\tau_n^2 I_n)}{\sum_{n=1}^{3}(\tau_n\, I_n)}\ (ps)$$

Text S2 The process of photocatalytic degradation of ODA

The degradation experiment uses a xenon lamp (Perfectlight, China) as light source with a power of 300 W, equipped with a filter (CUT 400) to ensure the output of visible light (λ ⩾ 400 nm), The emission spectrum of xenon lamp is shown in **Supplementary Fig.** 17(a). The distance between the light outlet and the liquid surface is 10 cm. Magnetic stirring (at a speed of 400 r/min) is used to ensure sufficient suspension of the photocatalyst, and the temperature of the degradation system is maintained at 25°C by circulating water. Before illumination, stir in a dark box for 2 hours to ensure sufficient contact and adsorption equilibrium between the catalyst and the solution. Then turn on the light source and start degrading. Samples were taken at different times and the concentrations of ODA was measured using a UV visible spectrophotometer. 3.0 mL of ODA solution to be tested was added into a volumetric flask; 2.0 mL of deionized water was added, followed by 1.0 mL of pH = 4 acetate-sodium acetate buffer solution, 2.0 mL of ethanol, and 0.5 mL of 0.5 mol/L sodium hydroxide solution. The mixture solution was shaken well and left to stand for 5 min. Then, 0.50 mL of 0.05% methyl orange solution was added into the mixed solution and place into a quartz cuvette. The absorbance of the solution to be tested was measured at 463 nm with the absorbance of deionized water as baseline. The standard curve of ODA concentration is shown in **Supplementary Supplementary Fig.** 17 (b).[2]

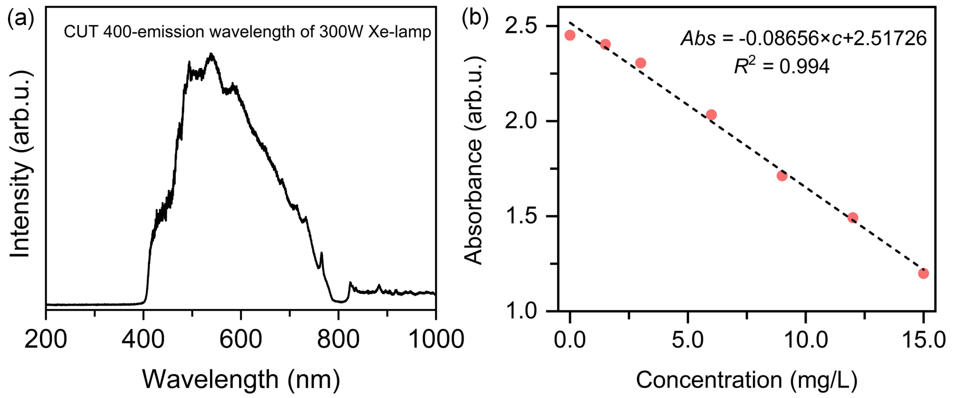

Supplementary Fig. 17 Light source and standard curve. (a) Xenon lamp emission spectrum, (b) ODA concentration standard curve.

Text S3 The process of photocatalytic degradation of DMP

The photocatalytic degradation process of DMP is the same as that of ODA, and its concentration testing method is 1.0 mL of DMP solution was mixed with 4.0 mL of pH = 4 acetate-sodium acetate buffer solution, 2.0 mL of 0.05% methyl orange solution, 15.0 mL of deionized water, and 5.0 mL of 1,2-dichloroethane. The mixture solution was shaken well and then left to stand for layering. Then, 8 mL of 2.0 mol/L hydrochloric acid solution was added into the organic phase in the lower layer of the layered solution following by shaken well and left to stand for layering. The upper aqueous phase of layered solution was transferred into a quartz cuvette for subsequent measurement. The absorbance of the solution to be tested was measured at 505 nm with the absorbance of deionized water as baseline. The standard curve of DMP concentration is shown in **Supplementary Supplementary Fig.** 18. [2]

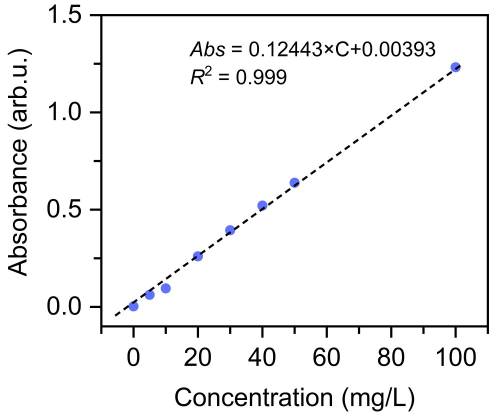

Supplementary Fig. 18 Standard curve. DMP concentration standard curve.

16. Degradation kinetics fitting curve

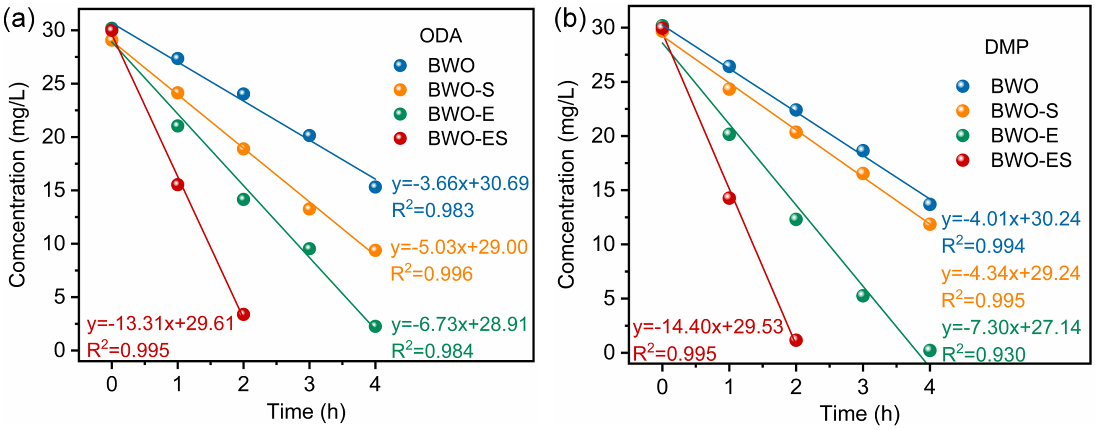

Supplementary Fig. 19 Kinetics fitting curve. Degradation kinetics fitting curve of (a) ODA and (b) DMP. [3]

17. Conversion of N element in ODA degradation process

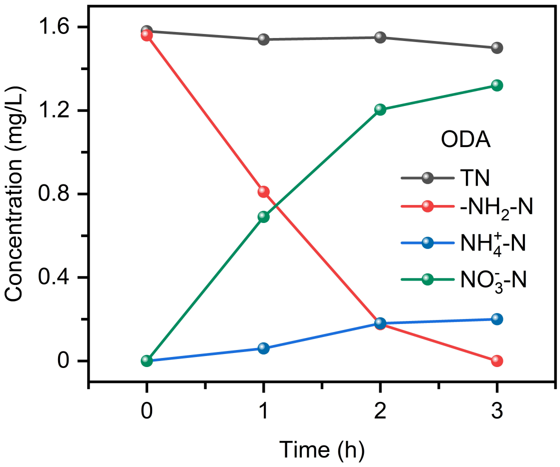

Supplementary Fig. 20 N element conversion in ODA. Conversion of N element in ODA degradation process.

18. TOC degradation curve

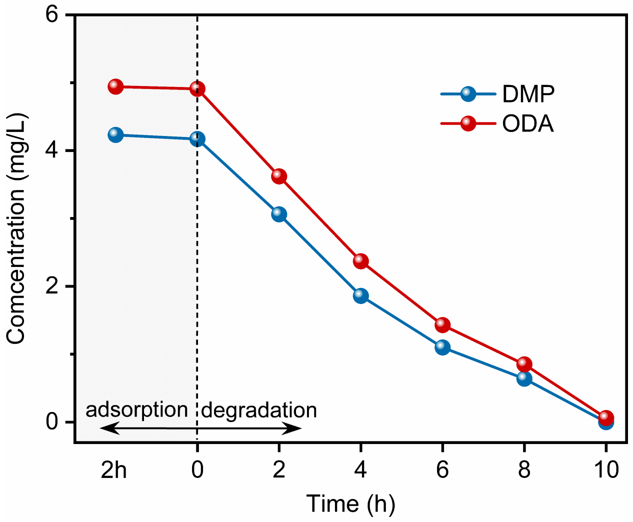

Supplementary Fig. 21 TOC removal. TOC degradation curve.

19. Infrared spectroscopy of catalyst surface in the degradation process of ODA

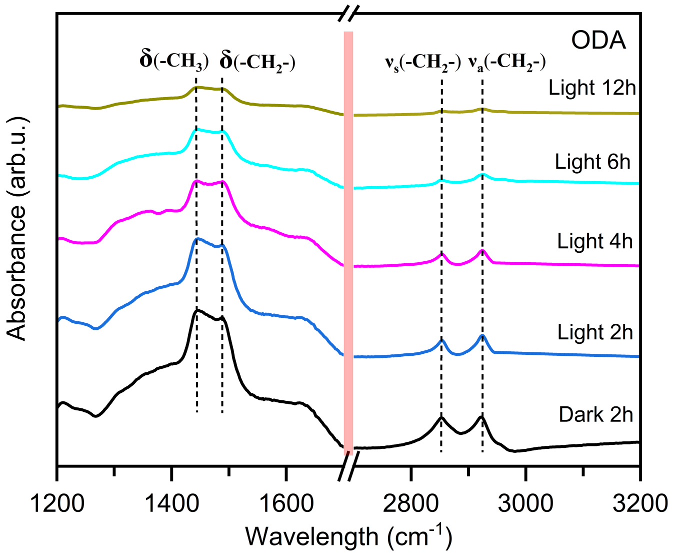

Supplementary Fig. 22 FTIR spectra. Solid phase infrared spectrum of catalyst surface during ODA degradation process (excluding BWO-ES substrate).

20. Quenching experiment of octadecylamine degradation

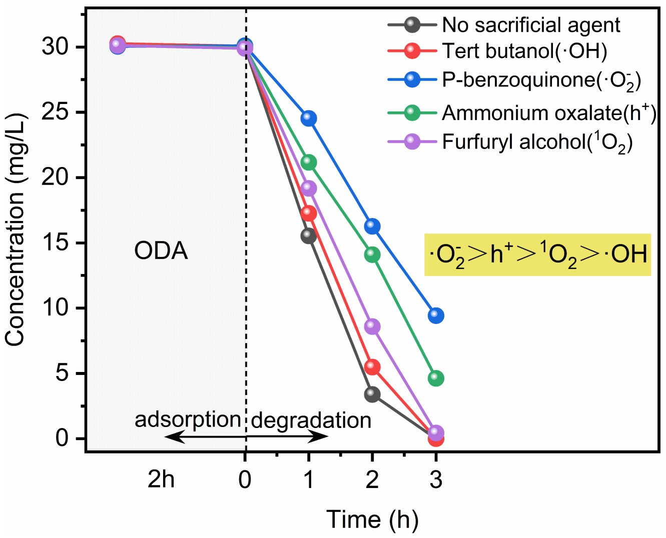

Supplementary Fig. 23 Quenching test. Quenching experiment of ODA degradation over BWO-ES. [4]

21. Quenching experiment Rate fitting

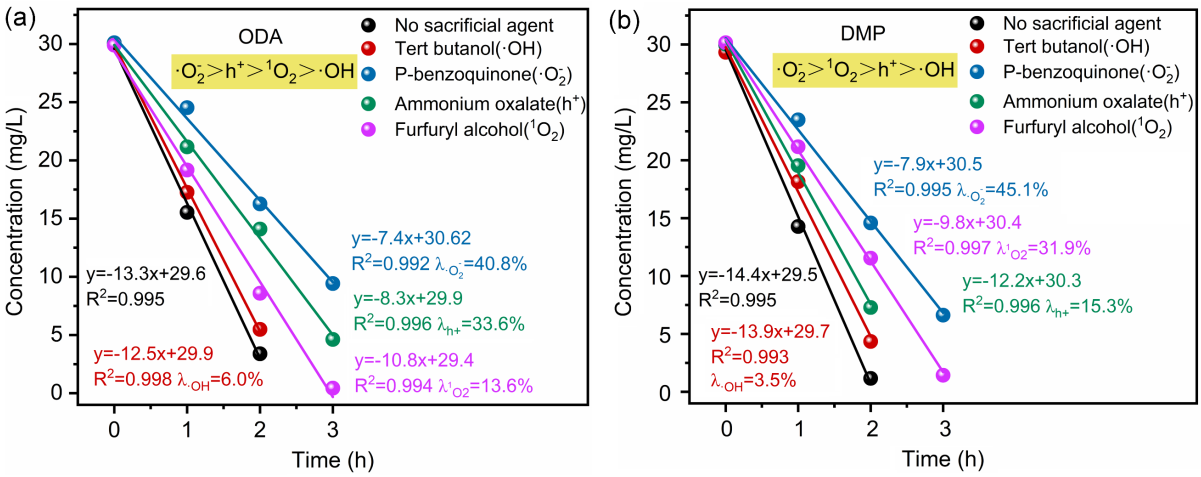

Supplementary Fig. 24 Quenching test kinetic fitting. Quenching experiment rate fitting of (a) ODA, (b) DMP degradation over BWO-ES.

By zero order kinetic fitting, degradation fitting curve ($y=kx+b$) was obtained under different quenching conditions, the formula is as follows:

$$\lambda\ (\cdot O_2^-) = \frac{K - K_{\cdot O_2^-}}{K}$$

$$\lambda\ (\cdot OH) = \frac{K - K_{\cdot OH}}{K}$$

$$\lambda\ (^1O_2) = \frac{K - K_{^1O_2}}{K}$$

$$\lambda\ (h^+) = \frac{K - K_{h+}}{K}$$

$$\lambda\ (other\ factors) = 1 - \lambda\ (\cdot O_2^-) - \lambda\ (\cdot OH) - \lambda\ (^1O_2) - \lambda\ (h^+)$$

where K is the degradation kinetics constant without quencher, while, $K_{\cdot O_2^-}$, $K_{\cdot OH}$, $K_{^1O_2}$, K_{h+} represents the degradation kinetics constants with the addition of p-benzoquinone, tert butanol, furfuryl alcohol, and ammonium oxalate. λ(X) represents the contribution ratio of each free radical to the degradation rate. [5]

22. EPR determination of ·OH generation

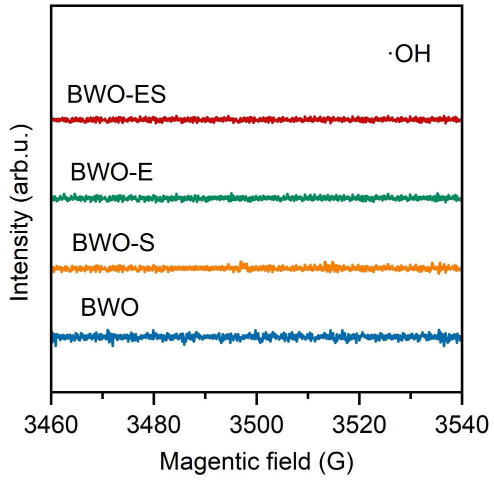

Supplementary Fig. 25 EPR spectra. EPR determination of ·OH generation under illumination conditions for Prepared materials.

23. Recycling stability of BWO-S and BWO-E

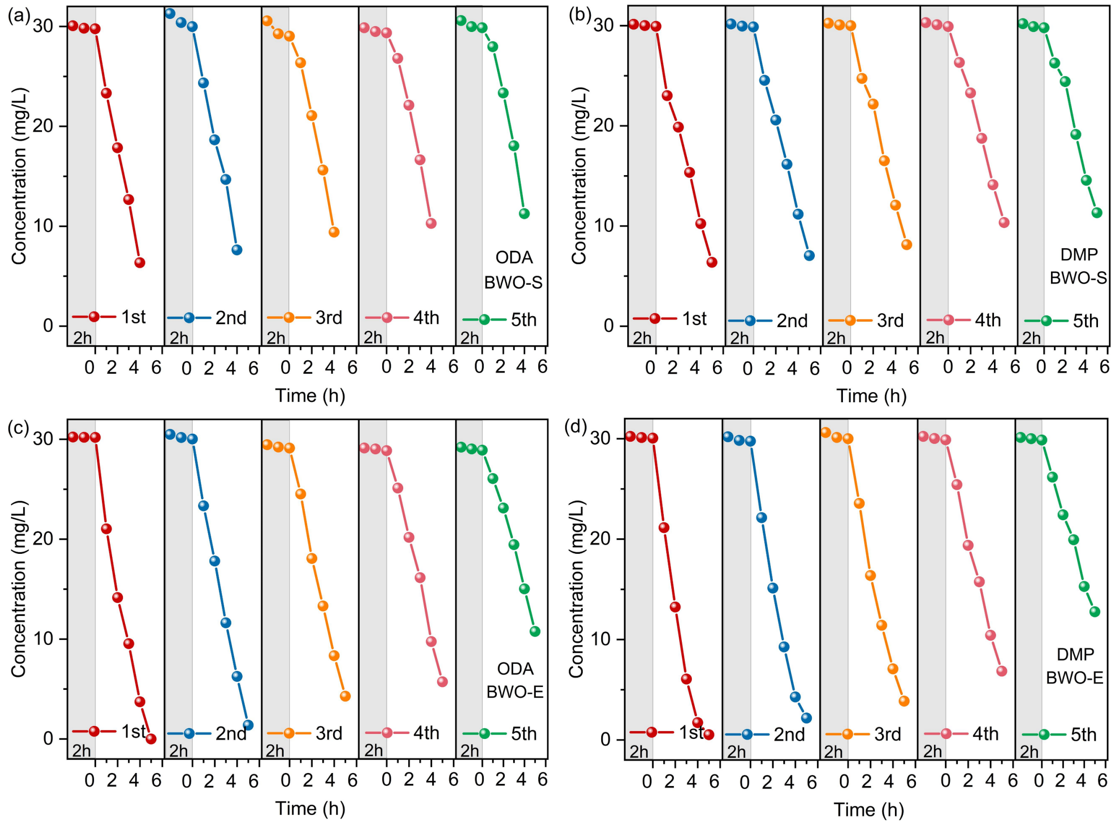

Supplementary Fig. 26 Stability test. Recycling stability of BWO-S and BWO-E samples during the degradation of (a), (c) ODA and (b), (d) DMP, respectively.

24. Recyclability of photodegradation of ODA

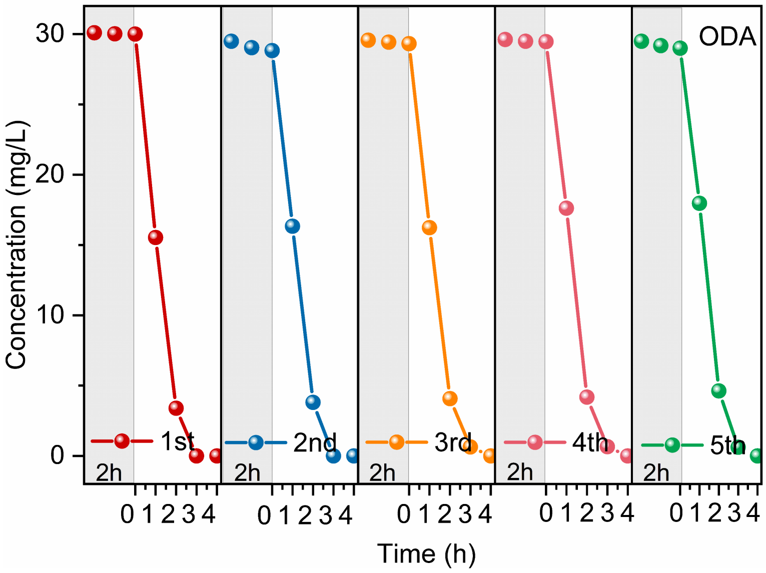

Supplementary Fig. 27 Stability test. Recyclability of photodegradation of ODA over BWO-ES.

25. Comparison of degradation performance of ODA and DMP between BWO-ES and reported materials

Supplementary Table 4 Comparison of the degradation performance of ODA and DMP by photocatalyst BWO-ES with materials reported in the literature.

Catalysts	Dosage	Degradation object	Volume/ Concentration	Degradation time	Degradation efficiency	Degradation medium	Light source	Refs.
TiO_2-A	120 mg	DMP	100 mL (50 mg/L)	3 h	100%	liquid phase	UV (280-380 nm)	[2]
BMS@TiO_2	100 mg	DMP	5 mg/g	16 h	92%	solid phase	UV (280-380 nm)	[6]
CPS (Cu_2O/SnO_2/PDA)	5 mg	DMP	5 mL (2 mg/L)	2 h	100%	liquid phase	Near infrared (808 nm)	[7]
WO/gCN-5	100 mg	DMP	100 mL (2 mg/L)	1 h	73%	liquid phase	Simulate sunlight	[8]
Fe-BMS	100 mg	DMP	14 mg/g	24 h	96%	solid phase	Simulate sunlight	[9]
UV photodegradation	-	DMP	50 mL (10 mg/L)	90 min	100%	solid phase	UV	[10]
BWO-ES	120 mg	DMP	100 mL (30 mg/L)	2 h	100%	liquid phase	Visible light (⩾400 nm)	This work
TiO_2-A	120 mg	ODA	100 mL (50 mg/L)	5 h	100%	liquid phase	UV (280-380 nm)	[2]
Fe-BMS	100 mg	ODA	50 mL (20 mg/L)	24 h	99%	liquid phase	Simulate sunlight	[9]
UV photodegradation	-	ODA	50 mL (10 mg/L)	80 min	100%	solid phase	UV	[10]
BWO-ES	120 mg	ODA	100 mL (30 mg/L)	3 h	100%	liquid phase	Visible light (⩾400 nm)	This work

26. UV-vis diffuse reflectance spectra

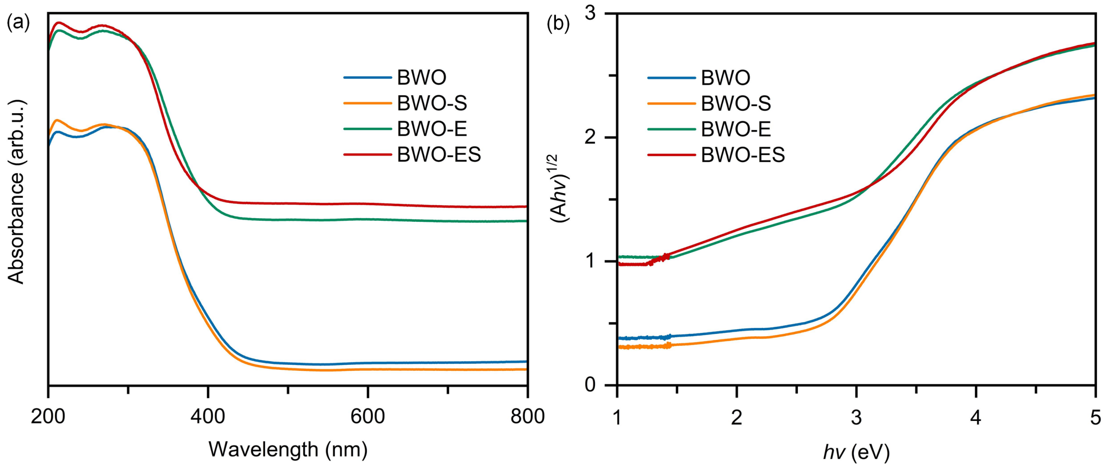

Supplementary Fig. 28 Light absorbance characteristics. (a) UV-vis diffuse reflectance spectra, and (b) plots of $(Ahv)^{1/2}$ versus photon energy (hv) of BWOs.

27. Calculated DOS

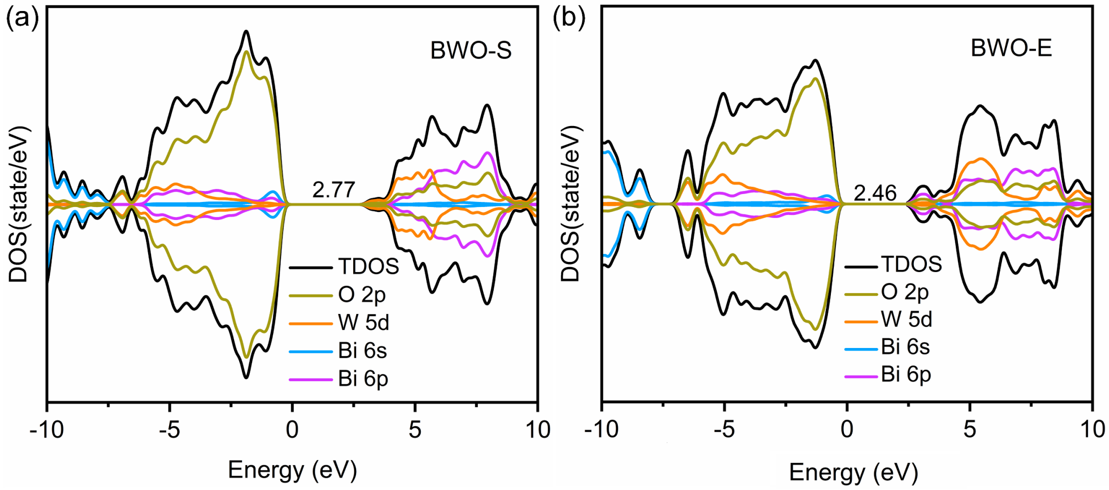

Supplementary Fig. 29 PDOS. Calculated DOS of (a) BWO-S, and (b) BWO-E.

28. PL spectra

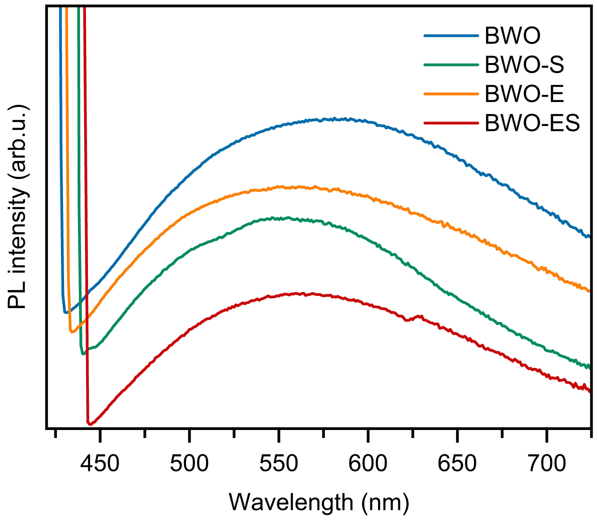

Supplementary Fig. 30 PL spectra of the samples. The PL spectra of BWO, BWO-S, BWO-E and BWO-ES samples.

29. The surface photovoltage spectra

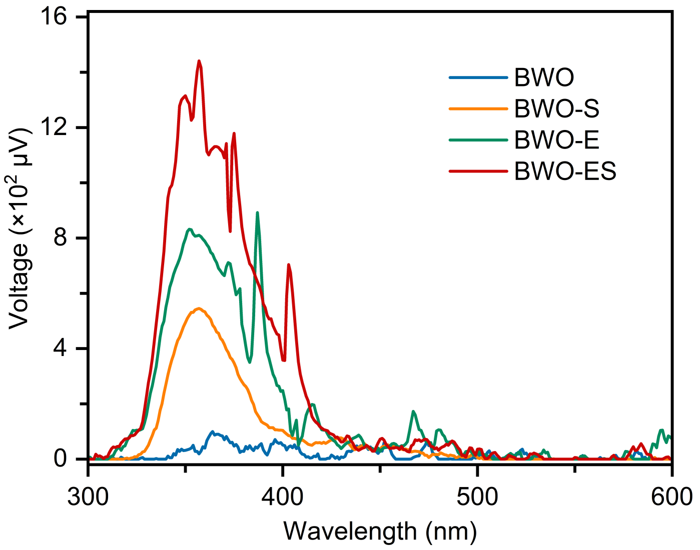

Supplementary Fig. 31 The surface photovoltage spectra. The surface photovoltage spectra of BWO, BWO-S, BWO-E and BWO-ES samples.

30. Model parameters of the photocatalysts based on EIS results

Supplementary Table 5 Model parameters of the photocatalysts based on EIS results.

	BWO	BWO-S	BWO-E	BWO-ES
R_1 (Ω·cm^2)	15.38	17.58	19.91	18.94
CPE1 (S·cm^{-2}·S^n)	4.285×10^{-4}	3.424×10^{-4}	1.469×10^{-4}	4.345×10^{-5}
n	0.6774	0.7072	0.7063	0.6268
R_{ct} (Ω·cm^2)	137.50	98.21	88.69	61.81
W_S (S·cm^{-2}·S$^{1/2}$)	3.295×10^{-3}	3.548×10^{-3}	3.777×10^{-3}	5.042×10^{-3}

R_1: Solution resistance; R_{ct}: Carrier migration resistance in semiconductor; CPE1: Constant phase angle element, representing interface capacitance; W_S: Weber impedance.

31. VB-XPS spectra of prepared materials

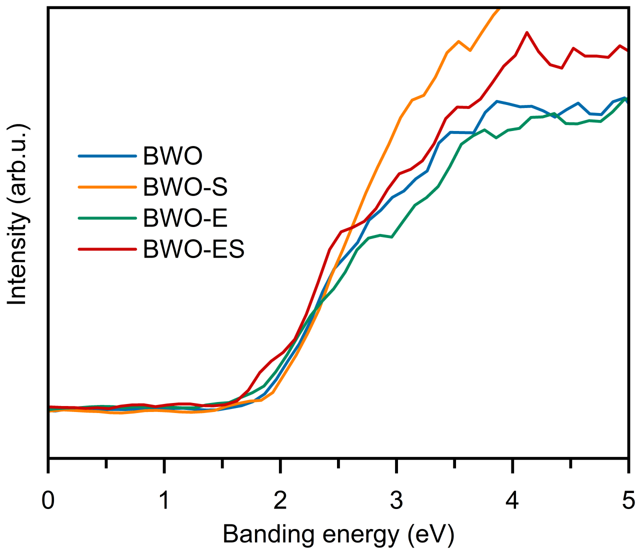

Supplementary Fig. 32 VB-XPS spectra. The valence band XPS spectra of BWO, BWO-S, BWO-E and BWO-ES samples.

32. The surface charge separation efficiency histogram

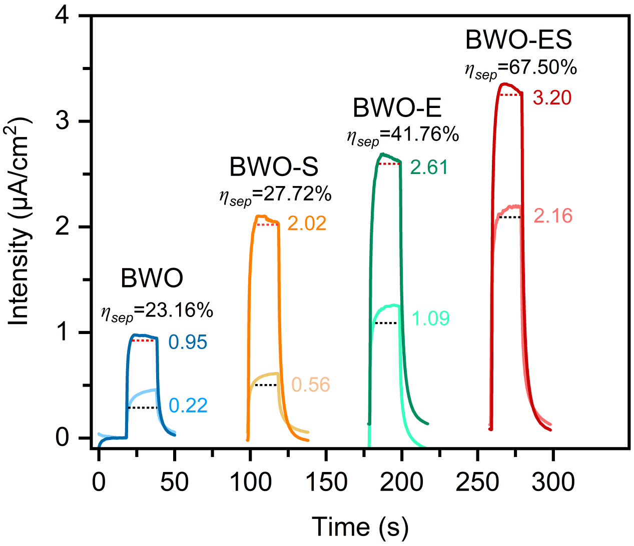

Supplementary Fig. 33 The charge separation efficiency. The charge separation efficiencies of BWO, BWO-S, BWO-E and BWO-ES.

The separation efficiency of photo generated e^- and h^+ was determined by adding the photo generated h^+ quencher Na_2SO_3. The formula is as follows:

$$\eta_{sep} = \left(J_{Na_2SO_4}/J_{Na_2SO_4+Na_2SO_3}\right) \times 100\%$$

（ $J_{Na_2SO_4}$ and $J_{Na_2SO_4+Na_2SO_3}$ represent the photocurrent densities measured in Na_2SO_4 electrolyte and Na_2SO_4/Na_2SO_3 mixed electrolyte solutions, respectively.）[11]

33. KPFM spectra

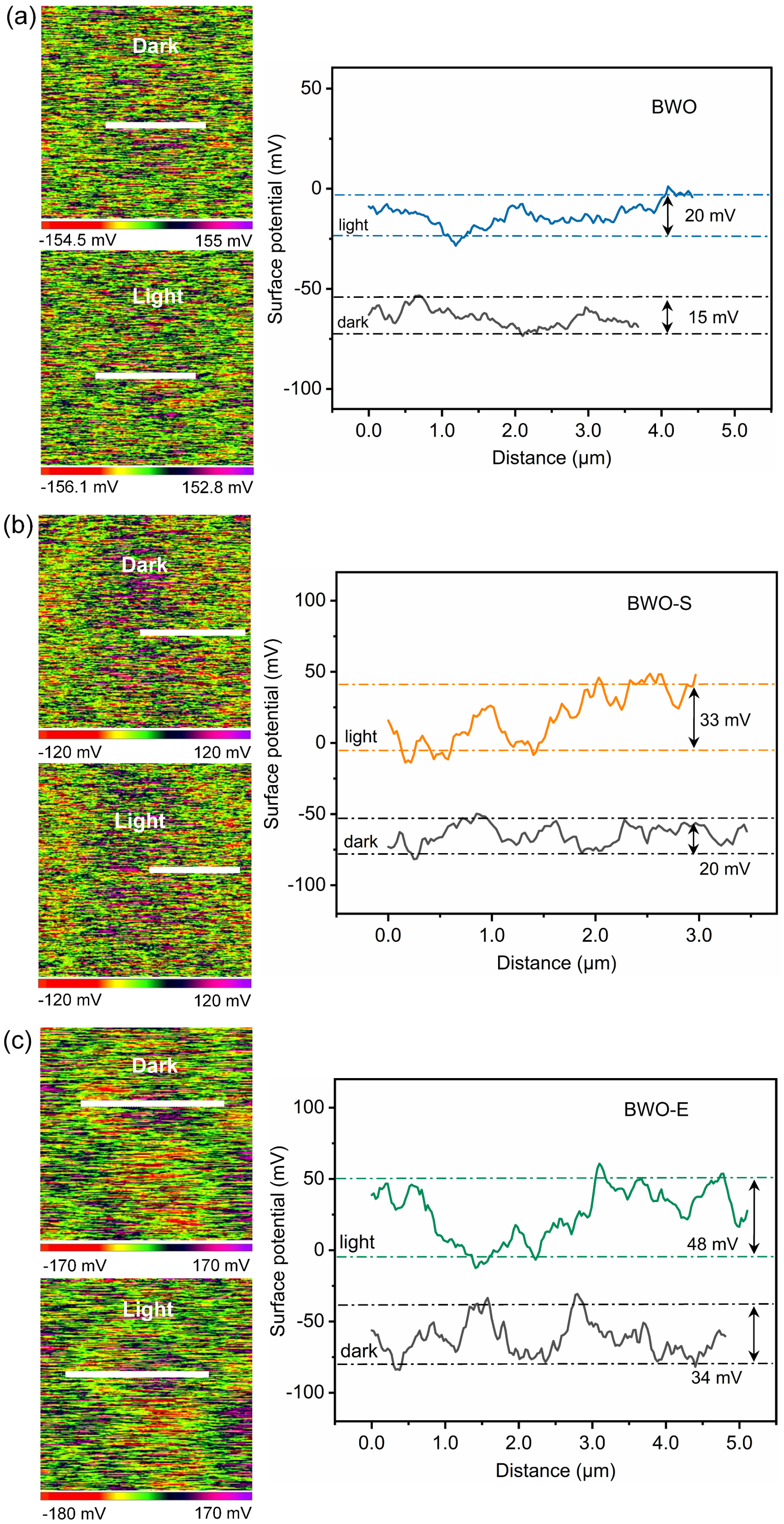

Supplementary Fig. 34 KPFM spectra. Kelvin Probe Force Microscopy (KPFM) spectra of (a) BWO, (b) BWO-S, (c) BWO-E.

34. The transient photocurrent response spectra

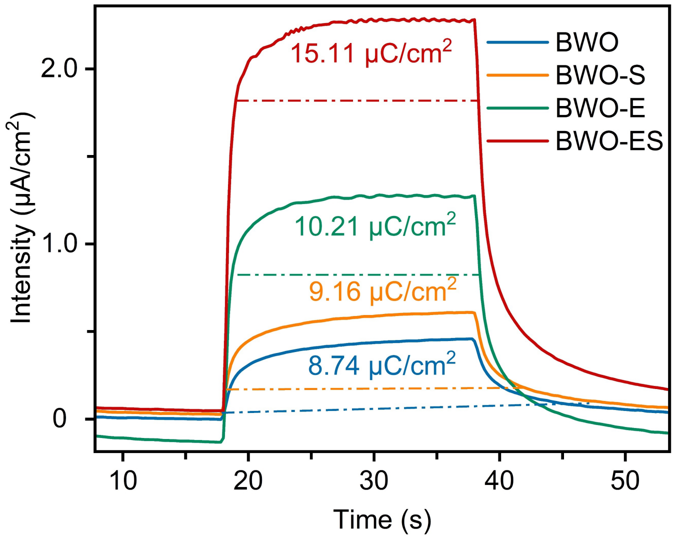

Supplementary Fig. 35 The transient photocurrent response. The transient photocurrent response spectra of BWO, BWO-S, BWO-E and BWO-ES.

According to Kanata-Kito model, the built-in electric field of materials can be calculated as follows:

$$E = \sqrt{\frac{-2V_S\rho}{\varepsilon\varepsilon_0}}$$

where E represents the intensity of the built-in electric field; V_S stands for the surface potential detected via KPFM; ρ is surface charge density, which will be obtained by the integral value of the current density; ε is the dielectric constant of Bi_2WO_6 (ε= 80 F m^{-1}); ε_0 refers to the vacuum dielectric constant (8.854×10^{-23} $J{\cdot}K^{-1}$). Thus, the V_S value is determined according to KPFM date (**Supplementary Fig.** 35) and the ρ value is determined according to **Supplementary Fig.** 36. [12]

35. Calculation of dipole moment

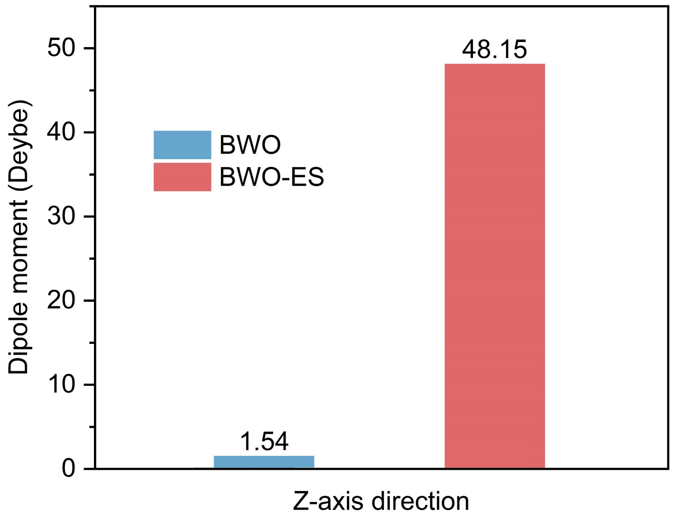

Supplementary Fig. 36 The calculated dipole moments. The calculated dipole moments along the Z axis for BWO and BWO-ES.

36 Toxicity analysis of ODA and DMP degradation products

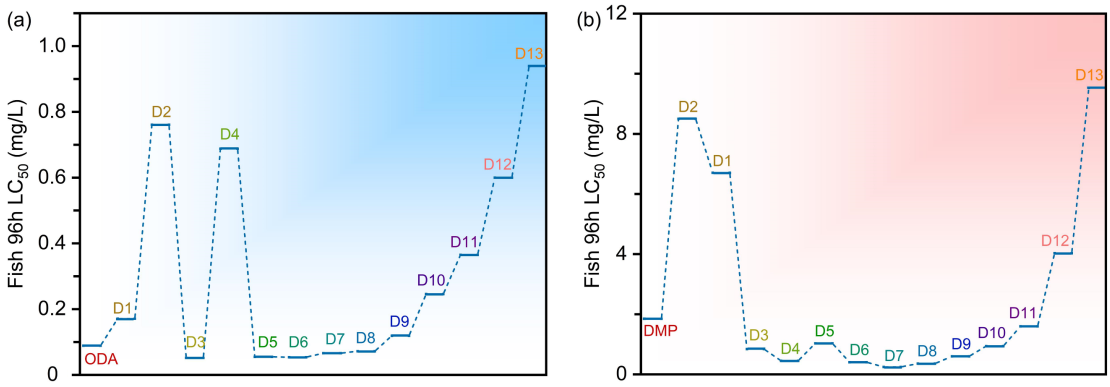

Supplementary Fig. 37. Toxicity evaluation. Acute toxicity of the fish 96h LC50, (a) ODA and (b) DMP degradation intermediates.

Evaluate through compTox chemicals Dashboard, v2.5.1. (United States Environmental Protection Agency.)

Supplementary Table 6 The forward and reverse flotation tailing wastewater samples composition sourced from a potash fertilizer production facility

	Sample	
	Forward flotation decompose slurry	Revese flotation tailing salt
pH	5.59	5.34
Conductivity (μS/cm)	151389	99702
Na^{+} (g/L)	73.13	52.55
K^{+} (g/L)	101.21	47.89
Ca^{2+} (g/L)	3.30	0.04
Mg^{2+} (g/L)	89.60	105.08
Cl^{-} (g/L)	257.35	298.44
Rb^{+} (mg/L)	99.60	0.38
Cs^{+} (mg/L)	2.00	0.36
CO_3^{2-} (mg/L)	441.00	1947.00
SO_4^{2-} (mg/L)	3.49	15.43
B (mg/L)	0.00	123.71
ODA (mg/L)	13.19	-
DMP (mg/L)	-	12.26
Salinity (%)	16.3	17.8

Text S4 Chemicals

Sodium tungstate ($Na_2WO_4 \cdot 2H_2O$, purity ≥ 98.0%), Octadecylamine ($C_{18}H_{39}N$, purity ≥ 97.0%）,Sodium acetate anhydrous (CH_3COONa, AR), Furfuryl alcohol ($C_5H_6O_2$, purity≥98.0%) were purchased from Aladdin (Shanghai, China). Bismuth nitrate pentahydrate [$Bi(NO_3)_3 \cdot 5H_2O$, purity≥99.0%], Ethylene glycol ($C_2H_6O_2$, purity≥99.5%), Sodium hydroxide (NaOH, purity≥ 99.0%), 1,2-Dichloroethane ($C_2H_4Cl_2$, purity≥99.5%), Methyl orange ($C_{14}H_{14}N_3NaO_3S$, AR.), Acetic acid ($C_2H_4O_2$, purity≥99.5%), Tert-Butanol ($C_{14}H_{10}O$, purity≥99.5%), p-Benzoquinone ($C_6H_4O_2$, purity≥99.0%), Ammonium oxalate [$(NH_4)_2C_2O_4 \cdot H_2O$, purity≥99.8%] were purchased from Macklin (Shanghai, China). Cetyltrimethylammonium bromide (CTAB) ($C_{19}H_{42}BrN$, purity ≥99.0%) was purchased from Rui Da Heng Hui Technology Development Co. (Beijing, China). hydrochloric acid (HCl) was purchased from Xilong Scientific Corporation Co. (Chengdu, China). Dodecyl morpholine ($C_{16}H_{33}NO$) was industrial grade.

Text S5 Crystal structure and morphology testing

1. X-ray diffraction (XRD): The crystalline structures of Bi_2WO_6 and defects BWO are identified by X-ray diffraction (D8 Discover, Bruker) using Cu-Kα irradiation (40 kV, 40 mA, 0.019° 2θ/s). Scan degree range from 5.010° to 79.98°.

2. Brunauer-Emmett-Teller surface area (S_{BET}): The porous characteristics and the specific surface area (S_{BET}) are determined by the N_2 adsorption/desorption method via a ASAP2460 instrument (micromeritics). All the samples are degassed at 200°C for 5 h prior to nitrogen adsorption measurements. The BET surface area is determined by the multipoint BET method using the adsorption data in the relative pressure (p/p_0) range of 0.06-0.30. Pore size distributions are calculated with the BJH method. The nitrogen adsorption volume at 77.35 K under the relative pressure (p/p_0) of 0.991 is used to determine the pore volume and average pore size. Equilibration interval 30s.

3. Scanning electron microscopy (SEM): The morphology of the mesoporous Bi2WO6 is observed by a field emission scanning electron microscope equipped with energy dispersive X-ray spectroscopy (SEM-EDX). SEM and SEM-EDX are carried out on SU8010 SEM (Hitachi/Oxford Instruments, UK) operating at 15 kV (resolution of 1.0 nm). The sample is dispersed in anhydrous ethanol and distributed on silicon wafers by ultrasound.

4. High resolution transmission electron microscopy (HR-TEM): The microstructure of the mesoporous Bi2WO6 is observed via transmission electron microscopy (JEM-F200, NEC/Oxford Instruments, UK) at 200 kV (resolution less than or equal to 0.10 nm).

5. Atomic force microscope (AFM): AFM testing was conducted using a Bruker Dimension Edge instrument (Germany). The catalyst was sonically dispersed in ethanol, and the supernatant was dropped onto mica flakes. The samples were then left to air dry naturally at room temperature before testing.

6. High-Angle annular dark field-scanning transmission electron microscope (HAADF-STEM): The instrument used is Thermo Fisher Spectra 300, equipped with a spotlight aberration corrector, with resolutions set at 50 pm (300kV) and 96 pm (60 kV). The electron energy loss spectrum (EELS) is measured using the electron energy loss spectrometer (Gatan-1065) equipped with it, with an energy resolution of 0.3 eV.

7. Fourier transform infrared spectroscopy (FT-IR): The functional groups of the Bi_2WO_6 were investigated by the transform infrared spectra (Thermo scientific, Nicolet iS50). The conventional KBr pellet technique was applied for the analysis of the adsorbed species on the surface of the Bi_2WO_6 photocatalyst. IR quality KBr (ANPEL Laboratory Technologies Inc, Shanghai, China) was used as the pellet matrix and diluent. Prior to characterization. The sample is first dried to remove moisture, and then mixed with dried potassium bromide powder in a mass ratio of 1:100, ground, and then compressed for testing, with a scanning range of 4000-400 cm^{-1}.

8. Laser Raman Microscopy Spectrometer (Roman): The testing instrument is Thermo Scientific (Nicolet DXR), and the material is tested for Raman shift from 50 cm^{-1} to 4000 cm^{-1}, under 532 nm laser excitation conditions.

Text S6 Elemental composition and valence analysis

1. X-ray fluorescence spectrometer (XRF): The analysis of sample element content was achieved through X-ray fluorescence spectroscopy (Rigaku, ZSX Primus II), which equipped with equipped with 4 kW, 30 μm thin window X-ray tube.

2. X-ray photoelectron spectroscopy (XPS): The elemental binding energy and valence state changes before and after defect construction in Bi_2WO_6 materials were investigated by the XPS (Axis Ultra DLD, Shimadzu), equipped with a monochromatized Al Kα (1486.6 eV) X-ray source. The in-situ argon plasma etching system was set to operate with the parameters of 2.0 kV accelerating voltage, beam density was 0.5 mA/cm^2, and incidence angle was 45°. Under such parameters, the etching rate of the in-situ argon plasma etching system on the Ta_2O_5 standard sample was 1.56 nm/s.

3. Positron Annihilation Technique (PAT): Positron annihilation technology reflects the electron density or momentum distribution information of positrons and is highly sensitive to atomic scale defects. Positron Lifetime Measurement Apparatus (ORTEC series) is equipped with ^{22}Na with a radiation intensity of 22 μCi, time resolution is 300 ps and the BaF_2 probe voltage is 2000 V.

4. Extended x-ray absorption fine structure (EXAFS): EXAFS measurements were conducted at the Shanghai Synchrotron Radiation Facility (SSRF) and Shanghai Institute of Applied Physics,Chinese Academy of Sciences (SINAP,CAS). The Si (111) bicrystal monochromator was used to filter X-ray beams, metal foil was used for energy calibration, and all samples were in transmission mode at room temperature. The test data was analyzed and processed using the Demeter software package.

Text S7 Photoelectrochemical property testing

1. UV-vis diffuse reflectance spectra (UV-vis DRS): Diffuse reflectance spectra are recorded by means of a Lambda 750s spectrophotometer (PerkinElmer) equipped with an integrating sphere, at room temperature in the spectral range 200-800 nm. A $BaSO_4$ pellet is used as a reference.

2. Kelvin probe force microscopy (KPFM): KPFM measures the surface potential of photocatalytic materials by applying an alternating voltage between the sample and the probe to detect the resulting electrostatic force. The instrument used is Bruker-Multimode 8 (Germany).

3. Piezoelectric force microscopy (PFM) tests were conducted using a Bruker Multimode 8 instrument. Test conditions maintained a room temperature of 28°C and relative humidity of 40%. Prior to testing, powder samples were pressed into thin discs approximately 5 mm in diameter, which were then scanned under varying voltage conditions.

4. Steady-state photoluminescence (PL) and time-resolved photoluminescence (TRPL) spectra: Photoluminescence and time-resolved photoluminescence spectra were recorded on an Edinburgh Instruments FLS1000 spectrofluorometer equipped with a xenon lamp (450 W), 60 W pulse xenon lamps, and a 980 nm diode laser as the excitation sources. The TRPL spectra was monitored using the FLS1000's time-correlated single-photon counting capability. Excitation was provided by an Edinburgh EPL-360 picosecond pulsed diode laser, the PL scanning range is 300-544 nm, TRPL scan time from 0 to 49.97 ns.

5. Electrochemical Characterization: The electrochemical measurements were performed on a Wuhan Coster electrochemical workstation (CS310H) with a conventional three-electrode cell. 2 mg catalyst was dispersed in a mixed solution (10 μL of 5% Nafion and 1.0 mL of ethanol), ultrasound for 15 minutes. Then, 100 μL suspension was dropped onto the ITO plate with 1 cm^2 and dried at room temperature, to be the working electrode. The counter electrode and the reference electrode were Pt plate and Ag/AgCl. The electrolyte was a 0.5 M Na_2SO_4 solution. The surface photocurrent and transient photocurrent response were measured under 300W Xe lamp ($\lambda > 365$nm), and the potential is 4 mV. Mott-Schottky plots were taken at frequencies of 3.0 kHz and a potential ranging from -1.1 to 0.1 V (vs. Ag/AgCl, pH = 7), respectively. Electrochemical impedance spectroscopy (EIS) was measured in a standard three-electrode system with the photocatalyst-coated ITO plate as working electrode, the Pt plate as counter electrode, and Ag/AgCl as reference electrode.

The amplitude of the sinusoidal wave was 10 mV, the testing rate is 10 mv/s and the frequency range was from 100 kHz to 0.1 Hz. The electrolyte is a mixture of potassium ferricyanide (1 mmol/L), potassium ferrocyanide (1 mmol/L) and KCl (0.1 mmol/L). The equipment used in the Surface photo voltage (SPV) testing process includes a 500 W xenon lamp (Swiss Franc XM500W, Beijing Xinda Technology Co., Ltd.), a dual particle monochromator (Zolix SP500), a lock-in amplifier with optical chopper (SR830-DSP) (SR540), and a sample cell. To ensure measurement accuracy and high signal-to-noise ratio, a special grounding wire PSS measurement system is equipped with a grounding resistance of less than 4 Ω.

6. Zeta potential analyzer: Zeta potential meter (Malvern Zetasizer Nano ZS90) was used to test the zeta potential of samples under pH=1,3,5,7,9,11 conditions.

Text S8 Investigation of degradation process

1. Electron spin resonance spectroscopy (ESR): Record the electron paramagnetic resonance spectrum using an electron paramagnetic resonance spectrometer (A300, Bruker, Germany). Record ESR spectra at room temperature using a quartz flat battery designed for solution. The conditions for ESR spectroscopy determination are as follows: microwave frequency of 9.853 GHz; The microwave power is 10.8 mW and the central magnetic field is 3510 G; The modulation amplitude is 1.0 G; The scanning range is 100 G, and the conversion time and time constant are both 1.250 ms; The scanning time is 19.456 seconds.

·OH detection: 2.0 mg of catalyst is ultrasonically dispersed in 10 mL of ultrapure water. 200 μL of the solution is taken and 200 μL of DMPO solution with a concentration of 50 mM is added. The solution is aspirated using a capillary tube, mixed and shaken well, and then placed in the machine for testing.

$^1O_2$ detection: Add 2.0 mg of catalyst to 10 mL of ultrapure water, disperse with ultrasound, transfer 200 μL of the solution, add 200 μL of a 50 mM concentration of TEMP solution, use a capillary pipette to aspirate the solution, mix and shake well, and then put it into the machine for testing.

$\cdot O_2^-$ detection: Add 2.0 mg of catalyst to 10 mL of methanol, disperse by ultrasound, transfer 200 μL of the solution, add 200 μL of DMPO solution with a concentration of 50 mM, use a capillary pipette to suck the solution, mix and shake well, and then put it into the machine for testing.

h^+ detection: Weigh 5.0 mg of catalyst and disperse it in 10mL of water. After 5 minutes of ultrasonic oscillation, take 200 μL of mixed solution and add 80 μL of Tempo solution (100 mM). After mixing and shaking well, put it into a capillary tube and then put it into a glass tube for machine testing.

2. Total organic carbon (TOC) and total nitrogen (TN): TOC and TN were recorded with a multi-N/C model TOC analyzer (Analytikyena, C/N3100, Germany).

3. Ion Chromatography (IC): Ionic chromatography (IC) systems separate charged particles from liquids and measure their concentrations based on the different retention times of ions. This experiment used a Thermo Scientific Dionex ion chromatography (ICS-1100) to analyze the concentrations of NH_4^+ and NO_3^- in the solution during the degradation process.

4. Gas Chromatography-Mass Spectrometry (GC-MS): The analysis of substances generated during

the material degradation process was conducted using Thermo Fisher Scientific (Triple Quadrupole GC-MS).

Toxicity analysis of organic compounds

The toxicity analysis of substances during the degradation process was conducted using complex Chemicals Dashboard v2.5.0 (Environmental protection agency of the United States)

Text S9 Calculation of energy band structure

The valence band (VB), conduction band (CB), and bandgap width (E_g) of different materials were calculated using density functional theory (DFT) using VASP code. The Perdew Burke Ernzerhof (PBE) functional in the generalized gradient approximation (GGA) is used to handle exchange correlations, while the projection enhanced wave pseudopotential (PAW) applies a kinetic energy cutoff of 500 eV to describe the expansion of the electron eigenfunction. The vacuum thickness is set to 25 Å to minimize interlayer interactions. The Brillouin zone integral is sampled from 7×7×7 Monk horst Pack k points centered on Gamma. All atomic positions are completely relaxed until the energy and force reach tolerances of 1×10^{-6} eV and 0.01 eV/Å, respectively. Using dispersion corrected DFT-D method to consider long-range interactions.

Text S10 Calculation of adsorption energy

Adsorption energy calculation: Simulation of the adsorption energy of oxygen molecules on BWO, BWO-ES specified crystal surfaces: Using the CASTEP module of the Materials Studio software package, the GGA (Generalized Gradient Approximation) method and PBE generalization for further structural optimization of the BWO and BWO-ES crystal structure models as well as the (001) crystal faces of both. The structural model of the O_2 molecule was constructed and the structure optimization was carried out using the same method. Subsequently, models of O_2 adsorption on the oxygen vacancies of the (001) crystal faces of BWO and BWO-ES were created, and the adsorption energies were calculated from the final energies.

Text S11 Finite element simulation of surface local electric field intensity in materials

This study employs the Finite-Difference Time-Domain (FDTD) method by COMSOL Multiphysics 5.6 software to calculate the surface local electric field intensity within nanostructures. During the calculation process, set the x, y, and z directions as perfectly matched layer conditions to prevent non-physical scattering. In addition, in order to obtain accurate calculation results, the entire simulation area was divided into grids of 1×1×1 nm. Importantly, based on the composition element ratios and defect distribution determined by XRF, a simulated structure of the material was constructed, using a full field scattering field plane wave with a wavelength greater than 400 nm as the excitation light source vertically incident on the surface of the nanostructure. Finally, we use an electric field monitor to obtain the internal electric field distribution of the nanostructured material.

Text S12 Calculation of dipole moment

Density functional theory (DFT) calculations were performed using the CP2K program in conjunction with the Gaussian plane wave (GPW) method. Valence electron molecular orbitals were expanded using the DZVP-MOLOPT-SR-GTH basis set, with an energy cutoff of 600 Ry. All simulations employed the Perdew-Burke-Ernzerhof (PBE) exchange-correlation functional, supplemented by the DFT-D3 (BJ) method, which effectively accounts for van der Waals interactions.

References

1. Wang, Y. H. et al. Gradient Cationic Vacancies Enabling Inner-To-Outer Tandem Homojunctions: Strong Local Internal Electric Field and Reformed Basic Sites Boosting CO_2 Photoreduction, *Adv. Mater*. 35 (2023) 31.
2. Ma, L. et al. Photocatalytic degradation of octadecylamine and 4-dodecylmorpholine over titanium based photocatalyst: Activity and mechanism insights, *Chem. Eng. J.* 472 (2023) 144782.
3. Hoffmann, M.R. et al. Environmental applications of semiconductor photocatalysis, *Chem. Rev.* 95 (1) (1995) 69-96.
4. Lu, M. et al. CNT/Nafion functionalized Ti/SnO_2-Sb/β-PbO_2-CNT/Nafion composite electrode toward highly active and robust degradation of octadecylamine and 4-dodecylmorpholine in real high-salinity system, *Chem. Eng. J.* 493 (2024) 152716.
5. Jiang, W. X. et al. Highlight the plasma-generated reactive oxygen species（ROSs）dominant to degradation of emerging contaminants based on experiment and density functional theory, *Sep. Purif. Technol.* 330 (2024) 125309.
6. Song, Z. M. et al. Basic magnesium sulfate@TiO_2 composite for efficient adsorption and photocatalytic degradation of 4-dodecylmorpholine in brine, *Sci. Rep.* 14 (2024) 9315.
7. Guo, P. S. et al. Photo-fenton catalysis degradation of 4-dodecylmorpholine by heterogeneous microprocessor Cu_2O/SnO_2, *J. Environ. Chem. Eng*. 12 (2024) 114682.
8. Zheng, L. X. et al. Construction of direct WO_3/g-C_3N_4 Z-scheme heterojunction for degrading flotation agent effectively, *Ceram. Int*. 50 (2024) 38860-38870.
9. Song, Z. M. et al. Designing an iron-doped basic magnesium sulfate photocatalyst for wide spectral photo response and superior catalytic activity, J. Mater. Chem. A. 13 (2025) 11573-11584.
10 Liu, T.T. et al. Degradation mechanism of octadecylamine and 4-dodecylmorpholine in Salk Lake Brine under UV light, *J. Environ. Chem. Eng.* 13 (2025) 115401.
11. Li, J. et al. Giant enhancement of internal electric field boosting bulk charge separation for photocatalysis, *Adv. mater.* 28 (2016) 4059-4064.
12. Zhu X. L. et al. Enhancing Built-in Electric Fields via Molecular Symmetry Modulation in Supramolecular Photocatalysts for Highly Efficient Photocatalytic Hydrogen Evolution, *Angew. Chem. Int. Ed.* 63 (2024) 26.
