# Supplementary material for: Region-specific defect engineering of Bi2W1-xO6-γ induces nanoscale electric fields and surface active-sites for enhanced visible-light oxidation of salt-lake flotation agents
Source: Nat Commun. 2025 Nov 28;16:11296. doi: 10.1038/s41467-025-66466-5 (PMC12722310; doi:10.1038/s41467-025-66466-5)
Supplement: Supplementary file 2 — Description of Additional Supplementary Files [file 41467_2025_66466_MOESM2_ESM.pdf]

Description of Additional Supplementary Files

File Name: Supplementary Data 1

Description: Identification of the degradation intermediates of ODA by GC-MS.

File Name: Supplementary Data 2

Description: Identification of the degradation intermediates of DMP by GC-MS.
